# Strategies for Achieving Healthy Energy Balance Among African Americans in the Mississippi Delta

**Published:** 2007-09-15

**Authors:** Groesbeck P Parham, Isabel C Scarinci

**Affiliations:** University of Alabama at Birmingham; University of Alabama, Birmingham, Alabama

## Abstract

**Introduction:**

Low-income African Americans who live in rural areas of the Deep South are particularly vulnerable to diseases associated with unhealthy energy imbalance. The Centers for Disease Control and Prevention (CDC) has suggested various physical activity strategies to achieve healthy energy balance. Our objective was to conduct formal, open-ended discussions with low-income African Americans in the Mississippi Delta to determine 1) their dietary habits and physical activity levels, 2) their attitudes toward CDC's suggested physical activity strategies, and 3) their suggestions on how to achieve CDC's strategies within their own environment.

**Methods:**

A qualitative method (focus groups) was used to conduct the study during 2005. Prestudy meetings were held with African American lay health workers to formulate a focus group topic guide, establish inclusion criteria for focus group participants, select meeting sites and times, and determine group segmentation guidelines. Focus groups were divided into two phases.

**Results:**

All discussions and focus group meetings were held in community centers within African American neighborhoods in the Mississippi Delta and were led by trained African American moderators. Phase I focus groups identified the following themes: overeating, low self-esteem, low income, lack of physical exercise, unhealthy methods of food preparation, a poor working definition of healthy energy balance, and superficial knowledge of strategies for achieving healthy energy balance. Phase 2 focus groups identified a preference for social support-based strategies for increasing physical activity levels.

**Conclusion:**

Energy balance strategies targeting low-income, rural African Americans in the Deep South may be more effective if they emphasize social interaction at the community and family levels and incorporate the concept of community volunteerism.

## Introduction

The obesity epidemic of the late 20th century and its adverse impact on health are of global proportions (1-3). Obesity, a common manifestation of energy imbalance, is a major risk factor for the development of type 2 diabetes, hypertension, stroke, coronary artery disease, and cancer and cancer-related mortality (4-6). Energy balance is classically defined as the balance between energy taken in, generally by food and drink, and energy expended. Lifestyle behaviors strongly linked to obesity are characterized by low levels of physical activity (sedentary lifestyle) or high consumption rates of high-fat or energy-dense diets, or both (1). Despite overwhelming data supporting physical activity and dietary habits as important predictors of weight change in individuals (7-11), long-term weight loss is rarely maintained (12) in the present obesity-promoting environment of the United States (13). Particularly problematic is evidence that racial and ethnic minorities, individuals with low levels of income and education, and populations with high obesity prevalence rates are less successful in weight-loss programs (2,4,14,15). Initial success with culturally relevant weight-loss programs among African American women, however, have shown promising results (16), particularly when participants were involved in program design and implementation (17).

### Rural environments and obesity

African Americans living in rural areas are at high risk for poor health. In general, populations in rural areas of the United States smoke more, exercise less, have less nutritious diets, and are more likely to be obese than populations living in suburban areas (18). Approximately 75% of African Americans living in rural areas reside in communities in the Deep South (i.e., Alabama, Georgia, Louisiana, Mississippi, North Carolina, South Carolina, and Texas) that are characterized by poverty and low income, both predictors of poor health. For instance, of all working-age African American adults in the South, 40% lacked a high school diploma during 1999–2000 compared with 14.9% of whites (19). Although approximately 12% of rural whites lived in poverty in 1999, nearly three times as many rural African Americans did so (19). Among rural adults who held occupations that ensured a worker would remain in poverty (e.g., domestic workers, maintenance workers), 68% were African American and 43% were white (19). In the state of Mississippi, the site of our study, the adult obesity prevalence rate is 28.1%, the highest in the nation (20), thus making it a high-risk environment for death from cancers associated with unhealthy energy imbalance. In Mississippi, death rates from such cancers (e.g., postmenopausal breast cancer, colon cancer, prostate cancer, pancreatic cancer) (21) are higher than the national average (22).

African Americans have very high rates of overweight and obesity and excessive incidence and mortality rates of some cancers (23,24). They are also among minority groups less likely to meet recommended levels of physical activity, a lifestyle factor critical to altering cancer risks. Such high levels of insufficient physical activity and persistent disparities in cancer underscore a need for better dissemination and adoption of strategies to increase physical activity levels within these high-risk communities. According to the Centers for Disease Control and Prevention's (CDC's) *Guide to Community Preventive Services* (25), the evidence-based strategies to increase physical activity levels include the following: community-wide campaigns, individual behavioral change, social support in community settings, the creation or enhancement of access to places for physical activity, and point-of-decision prompts.

## Methods

### Research design

We held discussions with a group (n = 18) of African American lay health workers from the Mississippi Delta for the purpose of defining the physical activity levels and dietary habits of our target population (i.e., low-income African Americans who live in the Mississippi Delta)We held discussions with a group (n = 18) of African American lay health workers from the Mississippi Delta for the purpose of defining the physical activity levels and dietary habits of our target population (i.e., low-income African Americans who live in the Mississippi Delta). We then held a series of focus groups with representatives from our target population. The objective of the focus groups was to 1) understand the focus group participants' attitudes and opinions toward CDC's suggested physical activity strategies to achieve healthy energy balance and 2) elicit their suggestions on how to achieve CDC's suggested strategies within their cultural and environmental milieu. We used focus groups as a method of formal assessment because they provide researchers with rich insights into the realities defined in a group process, particularly the dynamic effects of interaction between expressed beliefs, attitudes, opinions, and feelings. This study was approved by the Institutional Review Board of the University of Alabama at Birmingham (UAB).

### Study setting

The Mississippi Delta consists of 20 rural counties along the Mississippi River, best characterized by excessive levels of poverty and predominant African American populations. The Delta has been referred to as a Third World country in the heart of America (26). Approximately 40% of African Americans in the Delta lack health care coverage, according to a conversation with Agnes Hinton, PhD, and co-principal investigator for the Deep South Network for Cancer Control, a member of the Special Populations Network funded by the National Cancer Institute (NCI) and based at UAB. All focus groups were conducted in Greenwood, Mississippi, which has a population of 18,425, an annual per capita income of $14,461 and a median age of 31.7 years. Approximately 65% of Greenwood's population is African American, one-third of which lives below the poverty level (27). Overall, 33.9% of Greenwood's total population, 28.8% of its families, 47.0% of those under the age of 18, and 20.0% of those 65 or older live below the poverty line (27). The lay health workers with whom we collaborated established the following criteria for our focus group participants: 1) low-income status, 2) African American, 3) male and female sex, 4) Mississippi Delta resident, and 5) minimum age of 19 (no maximum age).

### Community health advisors and research partners (CHARPs)

The lay health workers with whom we collaborated are known as community health advisors and research partners (CHARPs). Since 2000, the community-outreach activities of CHARPs have been coordinated by the Deep South Network for Cancer Control. CHARPs live and work in the communities they serve and often share the same dietary and physical activity lifestyles and health-risk factors as their constituents. Therefore, they represented a natural and logical collaborator for this investigative effort. All CHARPs participating in this study were low-income African American men and women aged 19 years or older.

### Procedure

#### Months 1 and 2: Development of a *Focus Group Topic Guide* and recruitment and consent of focus group participants

During the first month of the study, we developed the Focus Group Topic Guide. First we convened a meeting between study investigators and CHARPs to determine 1) the types of questions to be asked in the focus groups, 2) how the questions were to be asked, 3) who would lead the focus groups, and 4) where, when, and what time the focus groups would be held. By first consulting with the CHARPs, we hoped to avoid the mistake of superimposing our ideas of what is significant in a topic guide onto the ideas of the target population. We then convened two prestudy preparation meetings between study investigators and CHARPs. During the initial prestudy preparation meeting, study investigators presented the study’s objectives, aims, rationale, significance, and methods to the CHARPs. The presentation was followed by an open discussion. The second prestudy preparation meeting consisted of 1) an open-ended discussion focused on defining the sociocultural environment of low-income African Americans in the Mississippi Delta, 2) a presentation by study investigators of the overarching themes of CDC’s suggested strategies for increasing physical activity levels and healthy eating (25,28), 3) a discussion of recruitment and retention strategies for focus group participants, and 4) the determination of sex and age segmentation. During the second month, the study was advertised in the local media and throughout informal community networks (e.g., churches, social clubs) by the CHARPs.

#### Months 3 to 5: Focus groups

We divided focus groups into phases 1 and 2. The goal of the first phase was to better understand the target audience's perceptions of health and the factors associated with their eating habits and physical activity levels. The goal of the second phase was to examine participants' perceptions of the benefits and barriers of using the CDC *Community Guide*'s strategies for increasing physical activity levels. We segmented Phase 1 focus group participants by sex and age (men and women; aged 19–45 years; aged >45 years). We conducted 10 focus groups: three groups of women aged 19 to 45 years; three groups of men aged 19 to 45 years; two groups of women aged greater than 45 years; and two groups of men aged greater than 45 years.

We did not segment Phase 2 participants by age because the responses of the participants in the Phase I focus groups did not differ according to age. We conducted six focus groups during Phase 2, three with men and three with women. The moderator explained the consent form individually to each focus group participant, and an interviewer administered a one-page demographic sheet to each participant. Trained moderators from UAB led each focus group; one moderator directed the group, and another took detailed notes. Both moderators matched the sex and race of focus group participants (29,30).

### Data analysis

Each focus group consisted of approximately eight participants. Each session lasted approximately 90 minutes, with participants financially compensated ($30) for their participation. All sessions were tape-recorded and transcribed to assist in the coding of themes and concerns. Open-ended data from focus groups discussions were analyzed in two stages. First, two raters independently read the original transcript and identified themes central to areas of discussion both within and across groups. Independent interpretations were discussed, and raters jointly decided upon a final coding scheme. The raters categorized individual comments according to themes to determine the range and significance of related responses. The second stage of the analysis involved summarizing data within and across groups. This phase of the analysis also included how themes were interrelated.

## Results

### Discussions with CHARPs

*Overeating* was the most common theme that surfaced during discussions on the causes of obesity among low-income African Americans in the Mississippi Delta. For example, one discussion participant observed, "People in the Mississippi Delta are used to three pieces of pork chop instead of one piece of pork chop, or four biscuits instead of one biscuit." When asked about reasons for overeating, the discussion participants identified the following: 1) low self-esteem, 2) a way of coping with depression or loneliness, 3) compensation for what they did not have during childhood, 4) social and family gatherings as a tradition, 5) easy accessibility to buffets, and 6) food stamps. Participants stated that food stamps provide a lot of food, but they do not teach recipients how to cook, shop, or prepare food. Table 1 provides sample comments on each reason for overeating suggested during the discussions.

Another common theme on the causes of obesity was that *obesity and overweight are not perceived as a health concern*. Participants had the following comments: "You know, people got this saying about what their doctor says, which is that if you are fat and you get sick, you got some meat stored." "A lot of people feel that as long as they can get around and get up and do what they gotta do, it does not matter how big they are. If they can get around and do what they gotta do, they think they are not overweight."

The CHARPs expressed the belief that lack of healthy eating among African Americans in the Mississippi Delta is not due to lack of knowledge. For example, one participant remarked, "Well, I am just saying that we all know what a proper helping should be." They identified the following barriers to healthy eating: 1) food price, 2) family structure or lack of behavioral rules on eating within the household, 3) lack of parenting skills, and 4) lack of assistance from health care providers. Sample comments on these barriers are provided in Table 1.

The CHARPs participating in the discussions agreed that African Americans in the Mississippi Delta are sedentary. The main reason given for not engaging in physical activity was lack of motivation. The following comments were made: "Well, in my neighborhood we got that [walk trail], and when I am passing through I might see one person out there. I might come back through and not see nobody out there." "I done worked on the job all day and that is walking. I am not fixing to do it."

After the group discussions, the CHARPs were asked to assist in developing a topic guide designed to probe the following issues within the focus groups:

- Overeating and barriers to healthy eating
- Obesity and overweight and related diseases
- Physical inactivity
- Benefits and barriers of using the CDC *Community Guide*'s suggested strategies for increasing physical activity levels
- Knowledge levels on healthy eating and physical activity

The CHARPs recommended that focus groups should be segmented according to age and sex, predicting that the responses for the age and sex groups would differ from each other.

### Focus group demographics

Phase 1 focus groups consisted of 36 participants (18 women and 18 men). Most of the Phase 1 participants also took part in Phase 2. Phase 2 focus groups consisted of 53 participants (28 men and 25 women). Characteristics of Phase 2 participants were as follows: women were significantly older than men (mean [SD] age of women, 49.8 [13.7] years; men, 38.9 [17.6] years), but men and women did not differ significantly in number of years of education (mean [SD] for men, 11.2 [2.3] years; women, 12.8 [4.0] years), monthly income (mean [SD] for men, $1302 [$751]; women, $1361 [$1187]), marital status, or employment status. Approximately 50% of Phase 2 participants were single (57.7% of men and 45.8% of women), and 30% were married or living with a significant other (34.6% of men and 33.3% of women). Approximately 40% of participants were currently employed (39.3% of men and 40% of women).

### Focus group discussions: Phase 1

Table 2 provides a summary of the topics discussed during the Phase 1 focus group meetings as well as a sample of responses. When asked the meaning of *good health* and *healthy living*, the focus group participants provided varied responses, but the most common themes were 1) good diet 2) stress-free living, 3) independent living, and 4) having a positive self-image. Definitions of exercise included walking, walking after eating, and sit-ups before going to bed. Questions about patterns of eating evoked a common theme of favoring high-volume meals. The most common themes on barriers to healthy cooking were 1) the influence of the family on what was cooked and how it was prepared, 2) the cost of food, and 3) lack of knowledge. The most common definition of physical activity was *being in motion.* Participants seemed to have a general understanding of the relationship between physical activity and disease prevention.

### Focus group discussions: Phase 2

The themes identified in Phase 2 differed by sex. Table 3 provides a summary of themes and sample participant comments. Themes identified by female participants on potential strategies to promote physical activity included 1) comprehensive approaches rather than isolated strategies, 2) strategies that are implemented with community involvement, 3) personalized programs that meet individual needs but are implemented in groups, including families, and 4) programs implemented in church settings. Themes discussed by men included 1) group activities involving family members, 2) no need for personalized programs, and 3) income as a major barrier to physical activity.

## Discussion

Our discussions with lay health workers and Phase 1 focus group participants uncovered beliefs, attitudes, and ideas about diet, physical activity, and health that relate to the development of an unhealthy energy imbalance among low-income African Americans in the Mississippi Delta. The level of understanding of the meaning of health among this population, particularly as it relates to the concept of energy balance, is superficial. Our findings show a strong culture of overeating, in which there is tremendous pride. Food is even sometimes used as a form of self-medication for the depressed psychological moods associated with low self-esteem and loneliness. Our findings also reveal a lack of information on how to prepare healthy meals and how to increase physical activity in a resource-constrained environment. In addition, participants reflected a lack of a sense of empowerment to facilitate the changes that are needed to achieve healthy energy balance both personally and as a community. Phase 2 focus group participants voiced a preference for community-based physical activity facilities that were financially and physically accessible to everyone. While both men and women expressed preferences for comprehensive, family-based, and "buddy system" approaches to physical activity, they differed significantly on the value of personal trainers. Women favored them more than men; men found them acceptable if they were the same sex as the trainee and they helped the whole family.

### Recommendations

Programs designed to develop energy-balance interventions for rural, low-income African Americans in settings similar to those of the Mississippi Delta should pay particular attention to the message being delivered. On the basis of our interpretation of discussions with CHARPs and focus group participants the message should be evidence-based, culturally appropriate, and environmentally relevant. It should 1) emphasize healthy lifestyles *and* the value of medical technology (e.g., cancer screening, blood glucose checks, blood pressure assessments) the value of medical technology (e.g., cancer screening, blood glucose checks, blood pressure assessments); 2) highlight healthy eating as well as physical activity; 3) consider the depressed socioeconomic environment and low self-esteem that characterize the living conditions and psychology of the target population; and 4) capitalize on the tremendous community pride, geographical identity, and respect for the family unit (nuclear and extended). The focus groups did not invite the participants to offer reasons for low self-esteem. The lack of self-esteem may be due to poverty, lack of education, or other individual characteristics. At the community level, the collective memories of racial segregation and mistreatment, especially in the American South, may also affect levels of self-esteem among African Americans (31-34). Program designers must be careful to present energy-balance messages in such a manner that they will not be perceived as demeaning or derogatory.

The seminal work of Eng and Parker (35) should serve as a guidepost for addressing the lack of empowerment among rural, low-income African Americans in the South. In a similar population and the same region of Mississippi as ours, they demonstrated that including political dynamics in the definition of community allowed health promotion programs to assist people in empowering their communities as much as they assisted people in improving their health. They focused on the challenge of confronting a system with difficult community issues. After one year of their intervention, community members were competent in mediating with outside institutions and officials.

Energy balance strategies targeting low-income, rural African Americans in the Mississippi Delta may be more effective if they 1) consider the history, culture, and environment of the target population, 2) emphasize social interaction at the community and family levels, and 3) incorporate the concepts of community volunteerism and political dynamics.

## Figures and Tables

**Table 1:** Summary of Results, Discussion Among Lay Health Workers (n = 18) on Overeating and Barriers to Healthy Eating Among Low-Income African Americans in the Mississippi Delta, 2005

Topic	Sample Comments
**Reasons for overeating**	
Low self-esteem	[People who overeat] don't have interest in themselves.
A way of coping with depression or loneliness	It is like . . . if your husband or man doesn't come home or leaves you, you go eating. And that is the only way you can go to sleep. You get full.
Compensation for what they did not have during childhood	I just feel like a person gets into a mode: "I did not have it when I was a kid, and now I can get whatever I want. I got the money. I am going to go and buy whatever I wanna. "You see, Daddy used to say, "Oh, don't give them no two pieces of chicken. Give them one." Now you got five.
Social or family gatherings as a tradition	It's getting together and the food be so good, and you hate to put it down.
Easy accessibility to buffets	I used to go to the buffet and just because I paid seven dollars I tried to eat twelve dollars worth of food.
Food stamps	[People who overeat] buy all of this food and they just eat, eat, eat. [People who overeat] do not know how to prepare meals. They should be teaching people how to use [food stamps] properly.
**Barriers to healthy eating**	
Food price	Eating healthy is very expensive.
Family structure and lack of behavioral rules on eating within household	Most families do not sit at the table anymore. You eat everywhere but at the table. We have lost the family structure.
Lack of parenting skills	Children do not raise you. You raise the children. But now we got the children raising the parents.
Lack of assistance from health care providers	Because I can compare my doctor here with the doctors I go to in Jackson and Memphis. They aren't concerned about your weight as the other doctors. I think doctors are not doing their part in trying to help us.

**Table 2:** Summary of Results, Discussion Among Phase 1 Focus Group Participants (n = 36) on Perceptions of Health and Factors Associated With Eating Habits and Physical Activity Among Low-Income African Americans in the Mississippi Delta, 2005

Topic and Response	Sample Comments
**Meaning of *good health* and *healthy living***	
Good diet	Good health means good eating habits and staying away from junk foods.
Stress-free living	Healthy living means having a body that can endure stress, work, family and other activities as well as being able to laugh and not take things seriously.
Independent living	Being able to work and take care of oneself. Having a sound mind.
Having a positive self-image	Feeling good about oneself.
**Definitions of exercise**	
Walking, walking after eating, and sit-ups before going to bed	—
**Identification of most prevalent diseases in their communities**	
Diabetes, hypertension, stroke, heart attack, cancer, and obesity among children	—
**Patterns of eating**	
High-volume meals	I load up. I fill my plate up and I eat all that I put on it.
**Barriers to healthy cooking**	
Influence of family on what is cooked and how it was prepared	I know how to cook healthy, but my family won't let me cook healthy.
Cost of food	A lot of people in the Delta are not as monetarily stable as they would like to be.
Lack of knowledge	I don't know how to prepare a nutritious meal.
**How free time is spent**	
Two most common answers were *watching television* and *no free time*, followed by range of answers including *walk, exercise, running after grandchildren, yard work, sleep,* and *read.*	—
**Definition of physical activity**	
Being in motion	Making it to the store. Chasing the kids. Mowing the lawn.
**Relationship between physical activity and disease prevention**	
Generally well understood	It builds the muscles in your heart. It keeps your muscles extended, blood circulating, and your heart valves open and your lungs . . . keep all these valves open. It keeps you going and strengthens your bones. Helps to improve breathing.
**Frequency of exercise**	
Almost no one reported exercising every day	—
**Motivation for exercise**	
Varied	Looking nice. Sexual stamina. Maintaining good health.
**Resources in the community for exercising**	
Varied from inadequate to adequate	—

Dashes (—) indicate that sample comments do not apply.

**Table 3:** Summary of Results, Discussion Among Phase 2 Focus Group Participants (n = 53) on Potential Strategies to Promote Physical Activity Among Low-Income African Americans in the Mississippi Delta, 2005

Topic	Sample Comment
**Women**	
Comprehensive approaches better than isolated strategies	Should cover both nutrition and physical activity, and provide specific information [e.g., recipes, exercises]. People are tired of isolated efforts. Ineffective as a "stand alone."
Strategies for the whole community	These activities must be guided and supervised, with built-in social support from the community.
Personalized programs for individual needs (considering age, sex, and health problems) but implemented in groups, including families	It would be good to have a personal trainer for the family and have some family physical activity program.
Programs implemented at church settings	Churches would be a good venue — messages from the pulpit, group walks, competitions across churches.
**Men**	
Group activities involving family members	Make it a group activity; include children and whole families.
No need for personalized programs	People may be resistant to constructive criticism from a personal trainer. [Trainers of the opposite sex] may lead to jealousy.
Income as a major barrier to physical activity	Cost is an issue.
